# Supplementary material for: Large-scale forward genetics screening identifies Trpa1 as a chemosensor for predator odor-evoked innate fear behaviors
Source: Nat Commun. 2018 May 23;9:2041. doi: 10.1038/s41467-018-04324-3 (PMC5966455; doi:10.1038/s41467-018-04324-3)
Supplement: Supplementary file 1 — Supplementary Informatio [file 41467_2018_4324_MOESM1_ESM.pdf]

Supplementary information for:

Large-scale forward genetics screening identifies Trpa1 as a chemosensor for predator odor-evoked innate fear behaviors

Wang et al.

Includes:

Supplementary Figures 1-13

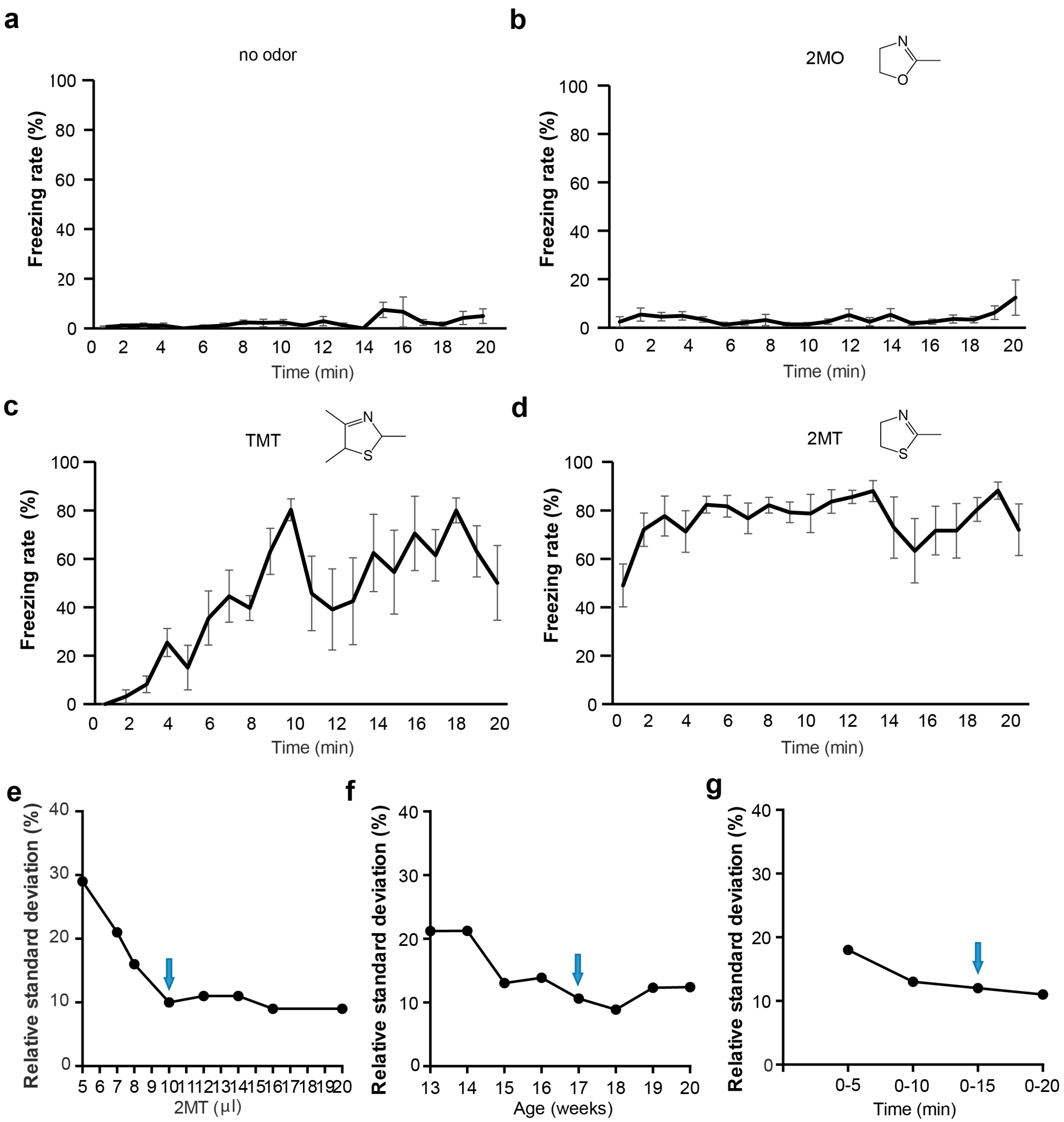

Supplementary Figure 1

Supplementary Figure 1: Development of a highly robust 2MT-evoked innate fear assay. (**a-d**) Graphs showing a time-course of freezing rates of wild type mice when exposed to regular air with no odor (**a**) or test odorant-containing gas with 10 ppm of 2MO (**b**), TMT (**c**), or 2MT (**d**) for 20 min in sealed test cages. Data are presented as mean ± SEM (n=8). (**e-g**) Graphs showing the relative standard deviation (RSD) values of the innate fear assay at different doses of 2MT exposure (**e**), ages of mice (**f**) and assay duration (**g**).

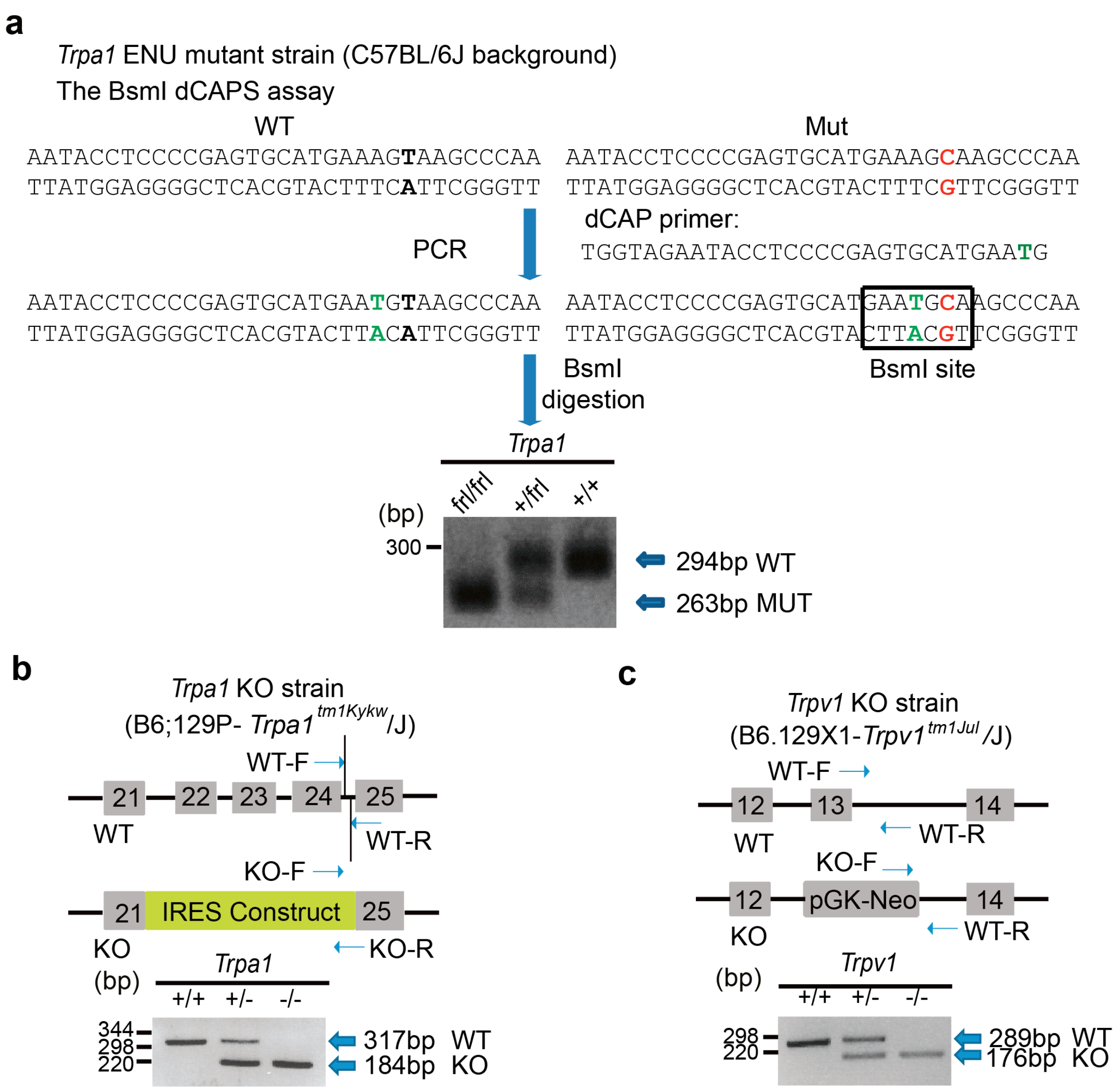

Supplementary Figure 2

Supplementary Figure 2. Genotyping of *Trpa1* and *Trpv1* mutant mice. (**a**) A schematic for genotyping *Trpa1 fearless (frl)* ENU mutant mice. dCAP primers are used to create a BsmI restriction site after PCR amplification of the *Trpa1*^{frl} mutant allele. After BsmI digestion, wild-type (WT, 294-bp) and mutant (MT, 263-bp) bands are resolved by Agarose gel electrophoresis. (**b-c**) A schematic for genotyping the *Trpa1* (**b**) and *Trpv1* (**c**) knockout (KO) mice using the PCR primers specified by the Jackson laboratory. Arrows mark the forward (F) and reverse (R) primers for amplifying the WT and KO alleles**.**

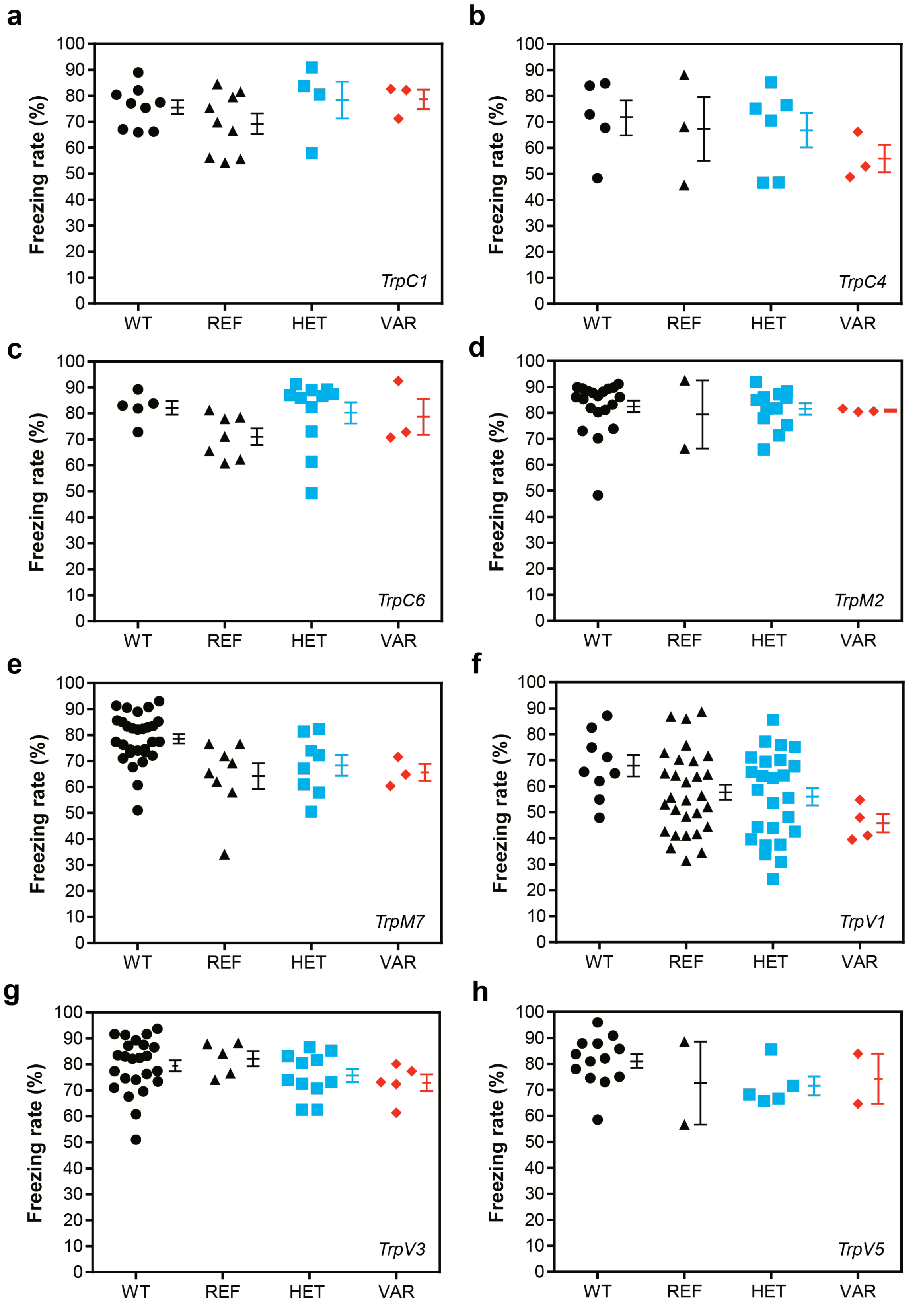

Supplementary Figure 3

Supplementary Figure 3. Multiple *Trp* mutant pedigrees exhibited normal 2MT-evoked innate freezing response. (**a-h**) Graphs of *Trpc1, Trpc4, Trpc6, Trpm2, Trpm7, Trpv1, Trpv3, Trpv5* mutant pedigrees that consist of reference (REF), heterozygous (HET), and phenovariant (VAR) individuals. A separate group of wild-type (WT) mice were included as controls for forward genetics screening every week.

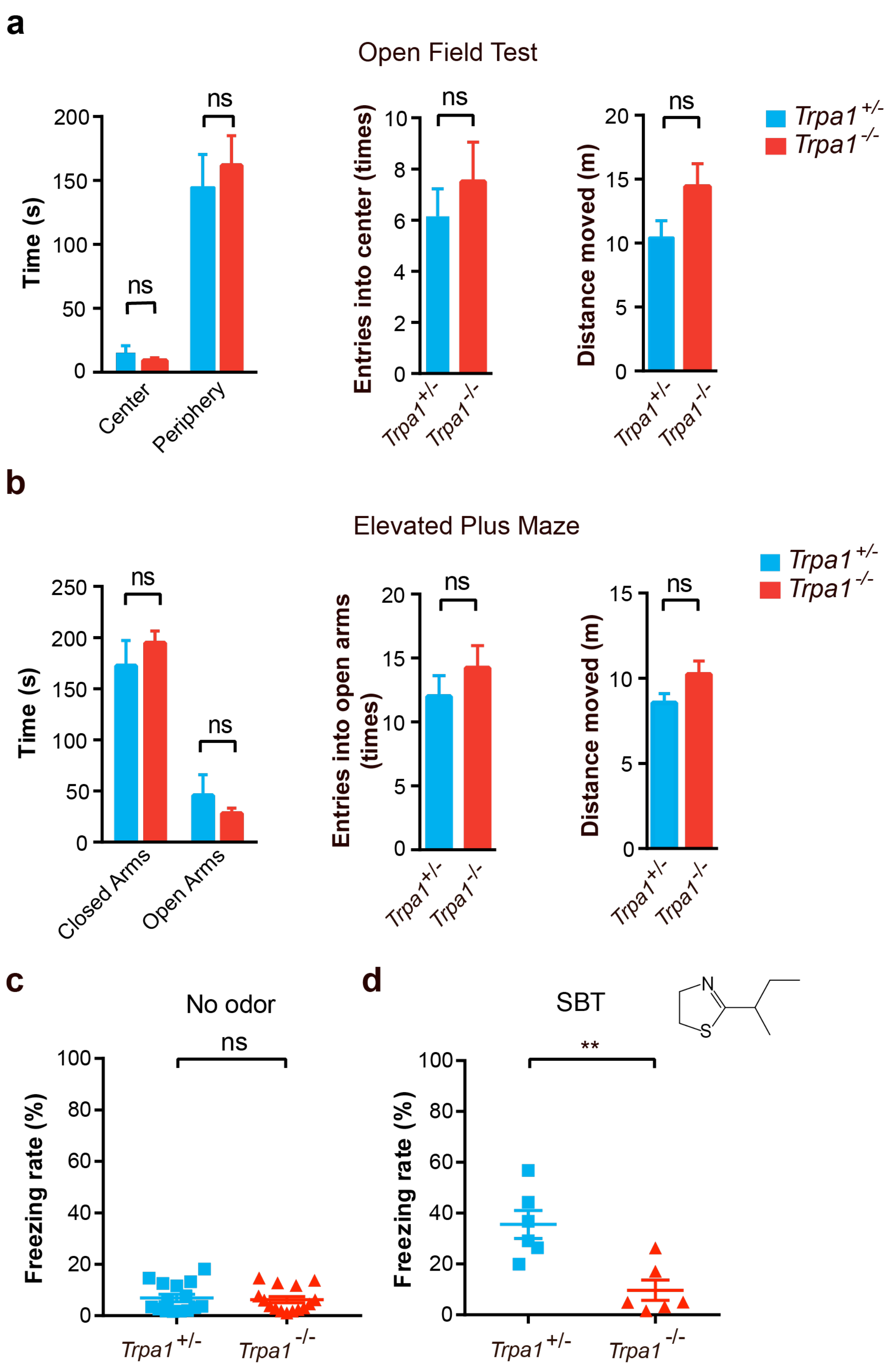

Supplementary Figure 4

Supplementary Figure 4. *Trpa1*$^{-/-}$ mice exhibited normal anxiety behaviors, but are defective for alarm pheromone SBT-evoked innate freezing. (**a**) The open field tests were performed on *Trpa1*$^{+/-}$ (n=13) and *Trpa1*$^{-/-}$ (n=14) mice. The total time that mice spent in the center and periphery of the open field was measured. The total distance traveled and the times of entry into the center were also quantified. Data are presented as mean ± SEM (Student's t-test, ns, not significant). (**b**) The elevated-plus maze tests were performed on *Trpa1*$^{+/-}$ (n=12) and *Trpa1*$^{-/-}$ (n=13) mice. The time that mice spent in the closed or open arms of the elevated-plus maze was measured. The total distance traveled and the times of entry into open arms were also quantified. Data are presented as mean ± SEM (Student's t-test, ns, not significant). (**c**) The baseline freezing rate of *Trpa1*$^{+/-}$ and *Trpa1*$^{-/-}$ mice were examined with no odor. Data are presented as mean ± SEM (n=15, Student's t-test, ns, not significant). (**d**) *Trpa1*$^{+/-}$ and *Trpa1*$^{-/-}$ mice were examined for SBT-evoked innate freezing behavior. Data are presented as mean ± SEM (n=6, Student's t-test, ** $P < 0.01$).

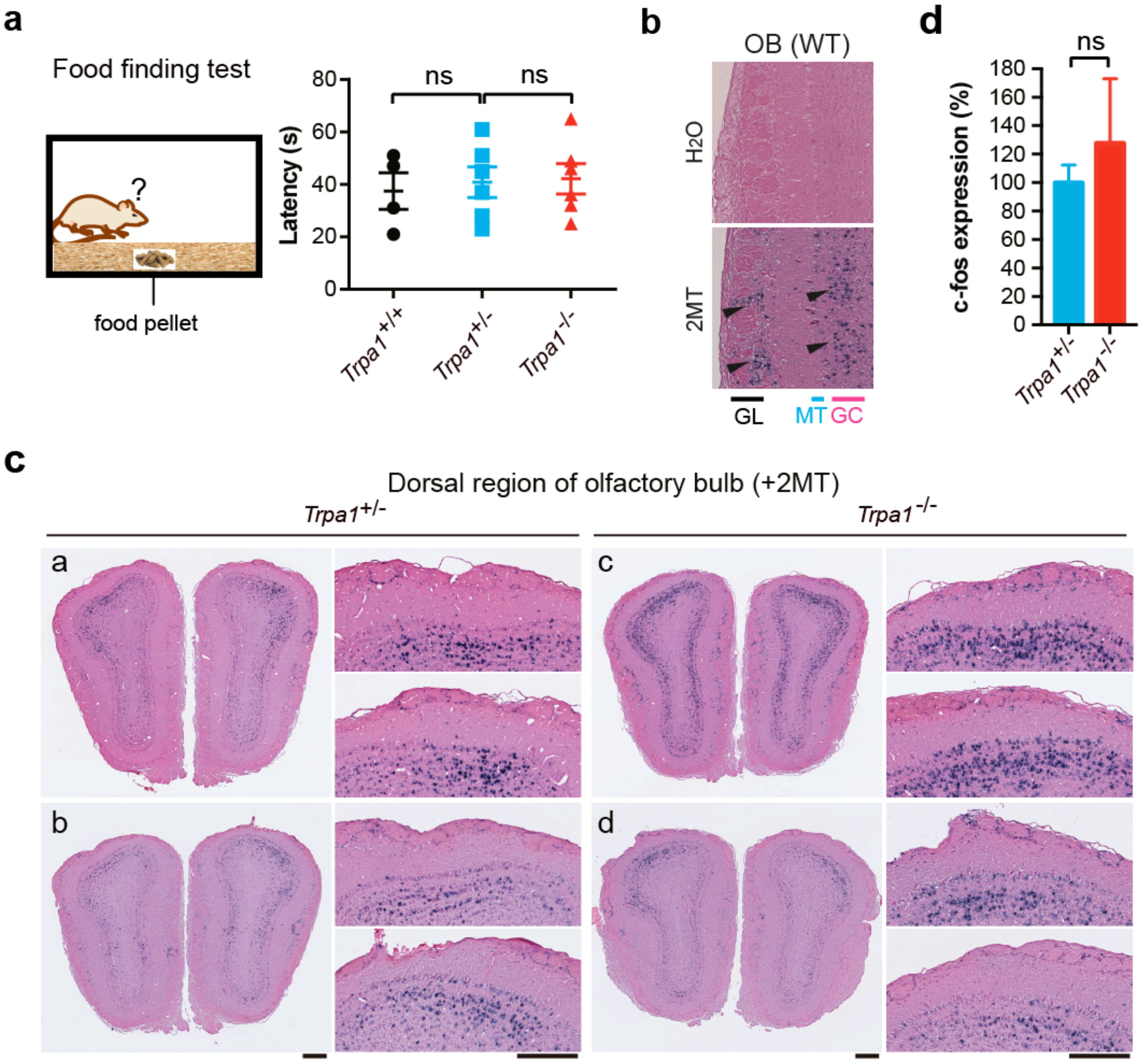

Supplementary Figure 5

Supplementary Figure 5. *Trpa1*$^{-/-}$ mice have a normal sense of smell. (**a**) After fasting overnight, *Trpa1*$^{+/+}$, *Trpa1*$^{+/-}$ and *Trpa1*$^{-/-}$ mice were placed into the cages with food pellets hidden beneath the bedding materials. The latency time was recorded from when the test mouse entered the cage to when it found the buried food. (**b**) Representative images showing specific *c-fos* ISH signals in the olfactory bulb (OB) of wild-type mice following H_2O or 2MT exposure. GL, Glomerular layer; MT, Mitral cell layer; GC, Granule cell layer. (**c**) Representative images showing specific *c-fos* ISH signals in the dorsal region of OB in *Trpa1*$^{+/-}$ (a and b) and *Trpa1*$^{-/-}$ mice (c and d) following 2MT exposure. Enlarged images of the dorsal OB are shown in the right panels. Bar: 200 μm. (**d**) Quantitative analysis of *c-fos* ISH signals in the dorsal OB of 2MT-exposed *Trpa1*$^{+/-}$ and *Trpa1*$^{-/-}$ mice. Data are presented as mean ± SEM (n=6, Student's t-test, ns, not significant). The relative measure (%) of *c-fos* signals of *Trpa1*$^{-/-}$ mice is normalized to those of *Trpa1*$^{+/-}$ mice.

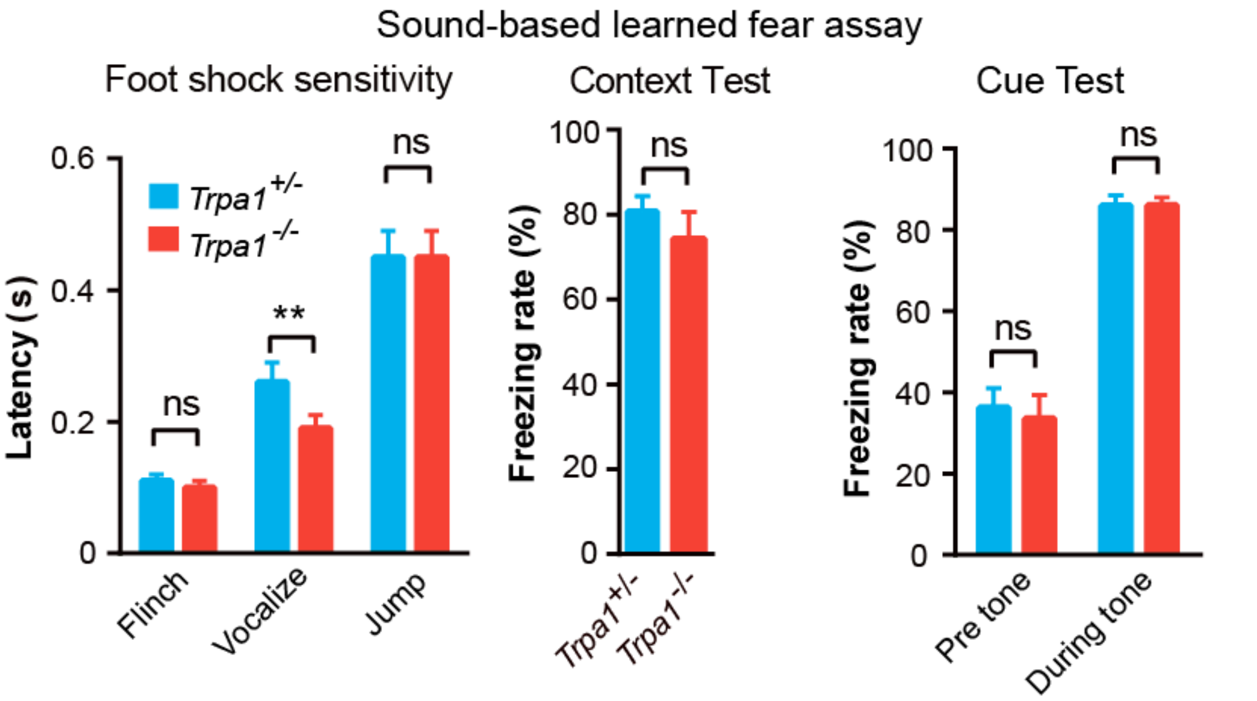

Supplementary Figure 6

Supplementary Figure 6. *Trpa1*$^{-/-}$ mice exhibit normal learned fear responses.

The sound-based learned fear assays were performed on *Trpa1*$^{+/-}$ (n=14) and *Trpa1*$^{-/-}$ (n=14) mice. As a measure of footshock sensitivity, the latency of flinching, vocalizing and jumping behaviors was measured (left). The freezing rates of *Trpa1*$^{+/-}$ and *Trpa1*$^{-/-}$ mice in response to the same training environment (middle, context test) or conditional auditory cue (right, cue test) were quantified. Data are presented as mean ± SEM (Student's t-test, ** $P < 0.01$, ns, not significant).

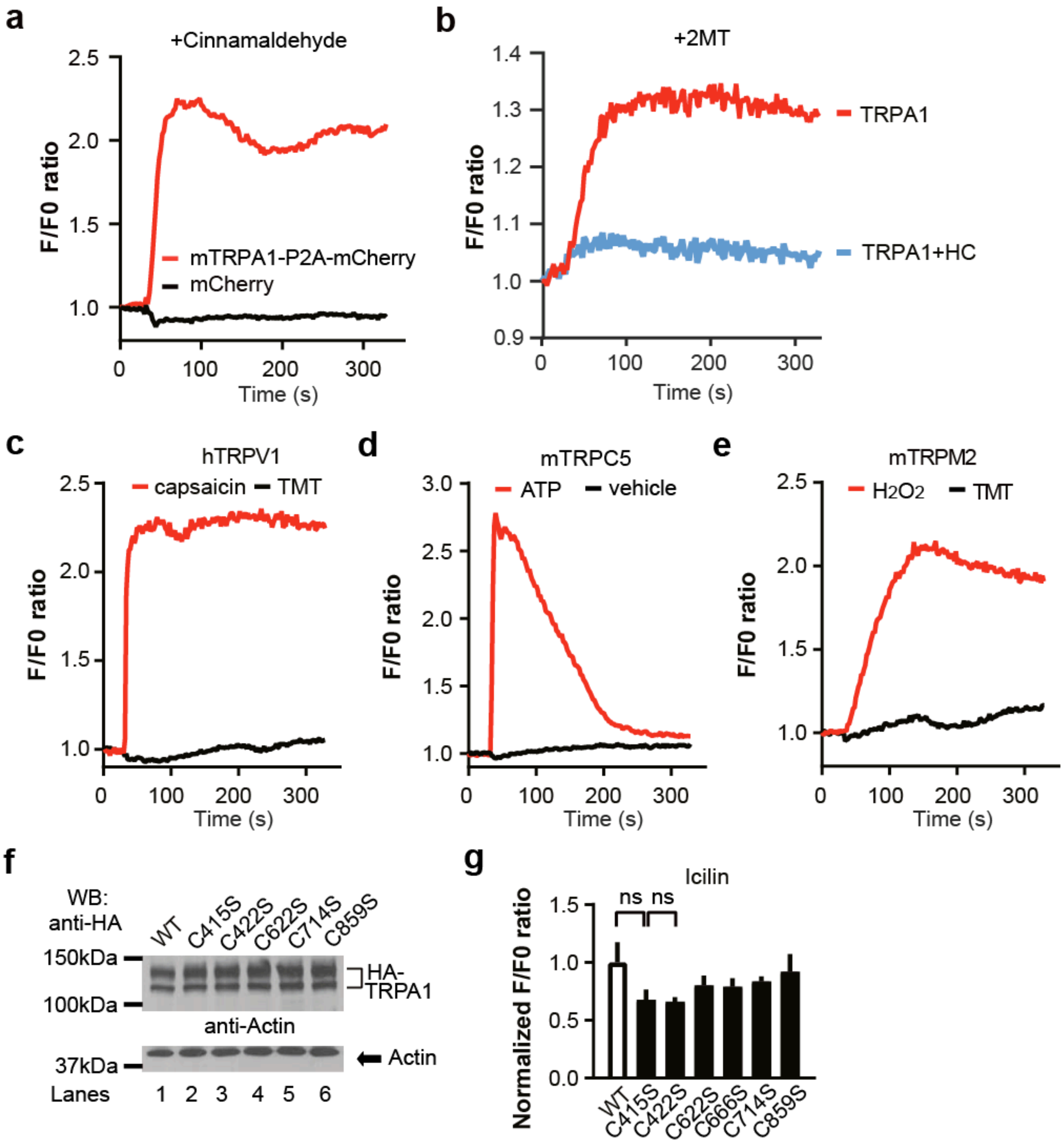

Supplementary Figure 7

Supplementary Figure 7. Trpa1 acts as a chemosensor for 2MT/TMT in heterologous cells. (**a**) Cinnamaldehyde (100 μM)-evoked Ca^{2+} response curves of HEK293T cells transfected with the Trpa1-P2A-mCherry or mCherry construct. (**b**) 2MT (100 μM)-evoked Ca^{2+} response curves of HEK293T cells transfected with the Trpa1-P2A-mCherry construct with or without HC-030031 (HC, 50 μM) treatment. (**c**) TMT (black, 100 μM) and capsaicin (red, 100 μM)-evoked Ca^{2+} response curves of HEK293T cells transfected with the human TRPV1 construct. (**d**) The vehicle (black) and ATP (red, 100 μM)-evoked Ca^{2+} response curves of HEK293T cells transfected with the mouse Trpc5 construct. (**e**) TMT (black, 100 μM) and H_2O_2 (red, 2.5 mM)-evoked Ca^{2+} response curves of HEK293T cells transfected with the mouse Trpm2 construct. (**f**) Western blotting was performed with anti-HA antibodies to compare the expression levels of wild-type (WT) and various Trpa1 mutant constructs. (**g**) Quantitative comparison of WT and mutant Trpa1 activities by icilin (500 μM)-evoked Ca^{2+} responses in transfected HEK293T cells. *Y*-axis, F/F0 ratio normalized to WT. Data are presented as mean ± SEM (n=3, Student's t-test, ns, not significant).

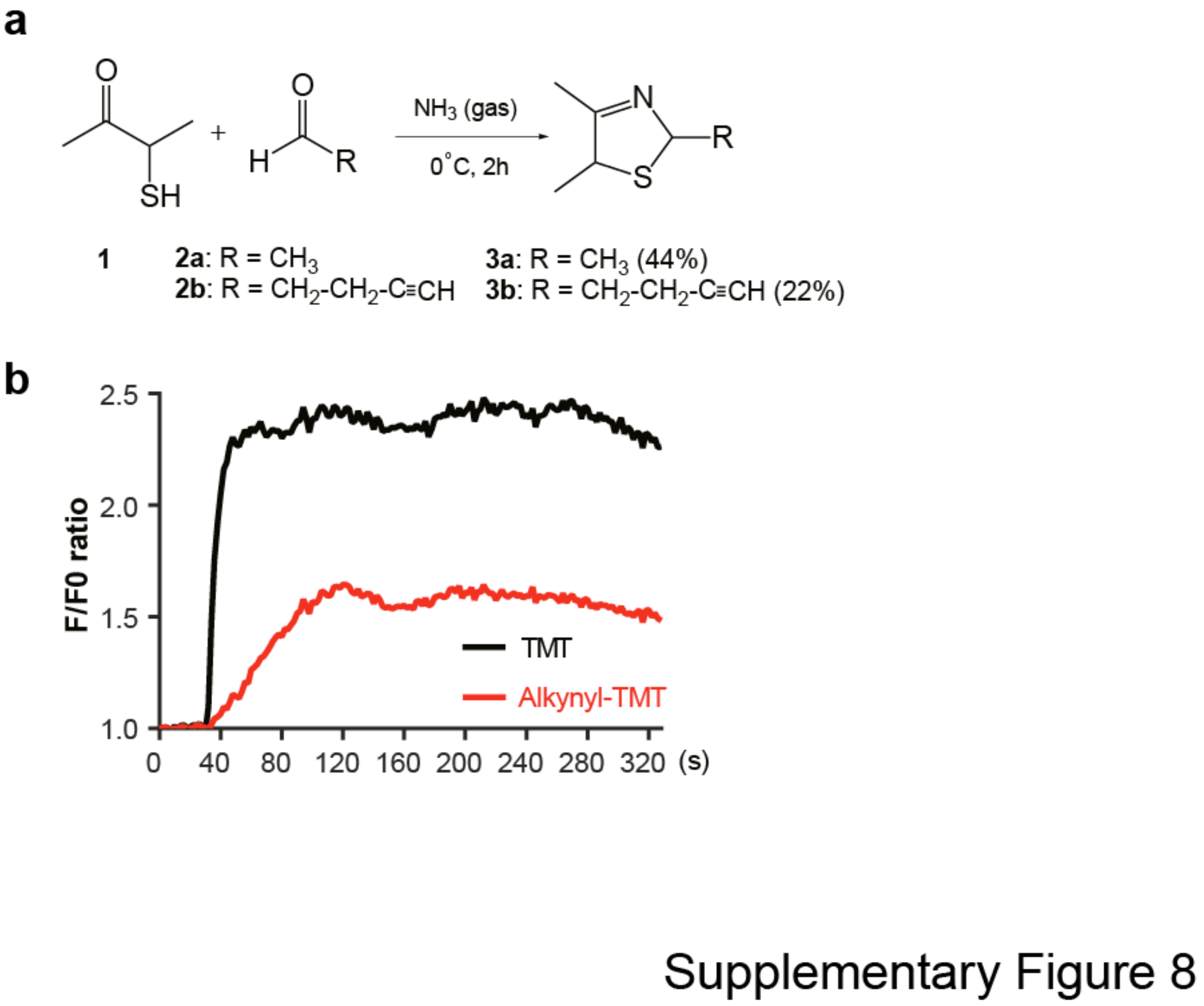

Supplementary Figure 8. Synthesis of alkynyl-TMT for click chemistry.

(**a**), A synthesis scheme of TMT (**3a**) and alkynyl-TMT (**3b**). (**b**) TMT (black, 100 μM) and alkynyl-TMT (red, 100 μM)-evoked Ca^{2+} response curves in transfected HEK293T cells.

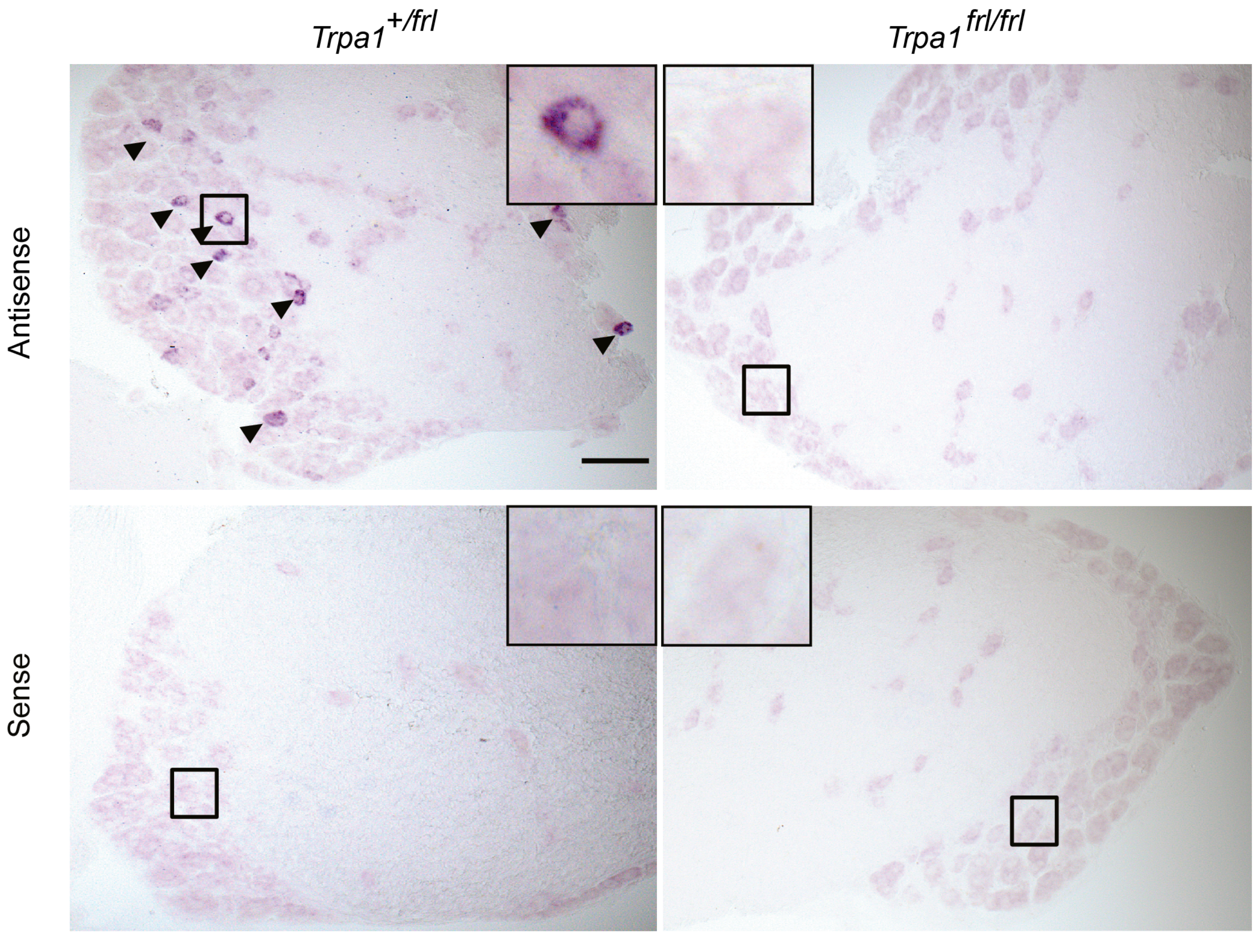

Supplementary Figure 9

Supplementary Figure 9. In situ hybridization of *Trpa1* mRNA in the trigeminal ganglion. Images showing that *Trpa1* mRNA is diminished in the trigeminal ganglia (TG) of *Trpa1*$^{frl/frl}$ mutant mice as compared to *Trpa1*$^{+/frl}$ mice. Antisense *Trpa1* probe (upper); Sense *Trpa1* probe (lower). Insets are magnified images showing specific *Trpa1* expression (purple). Bar: 50 µm.

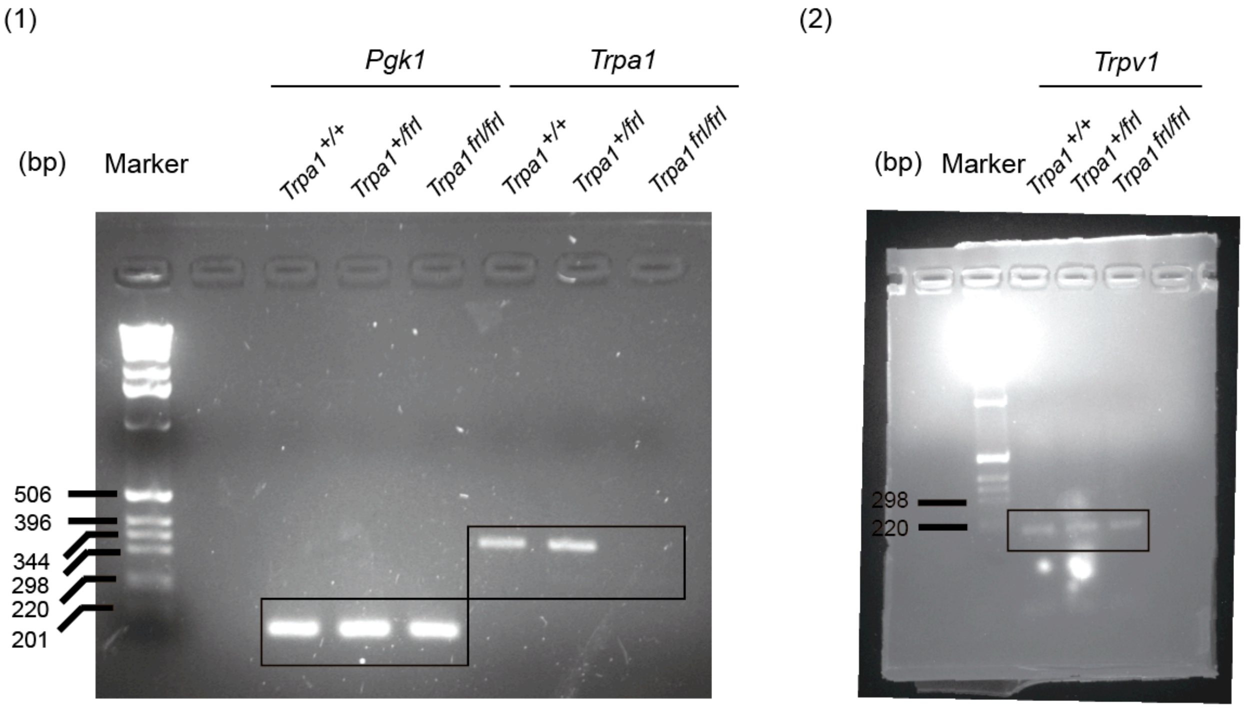

Supplementary Figure 10. Full unedited DNA gel images for Fig. 2f. Solid lines indicate areas shown in the main Figure.

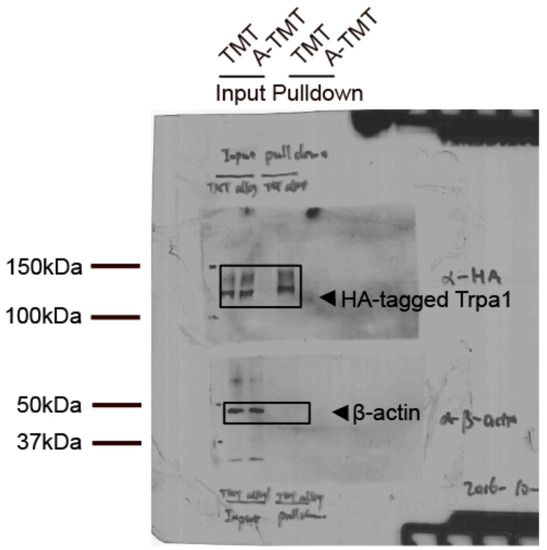

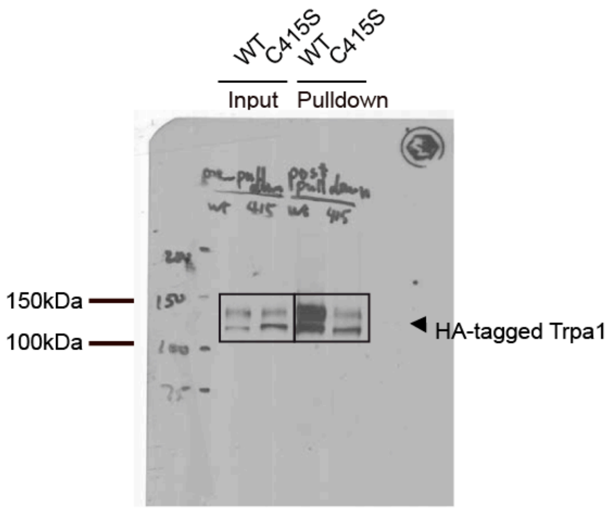

Supplementary Figure 11

Supplementary Figure 11. Full unedited DNA gel images for Fig. 5g, 5h. Solid lines indicate areas shown in the main Figure.

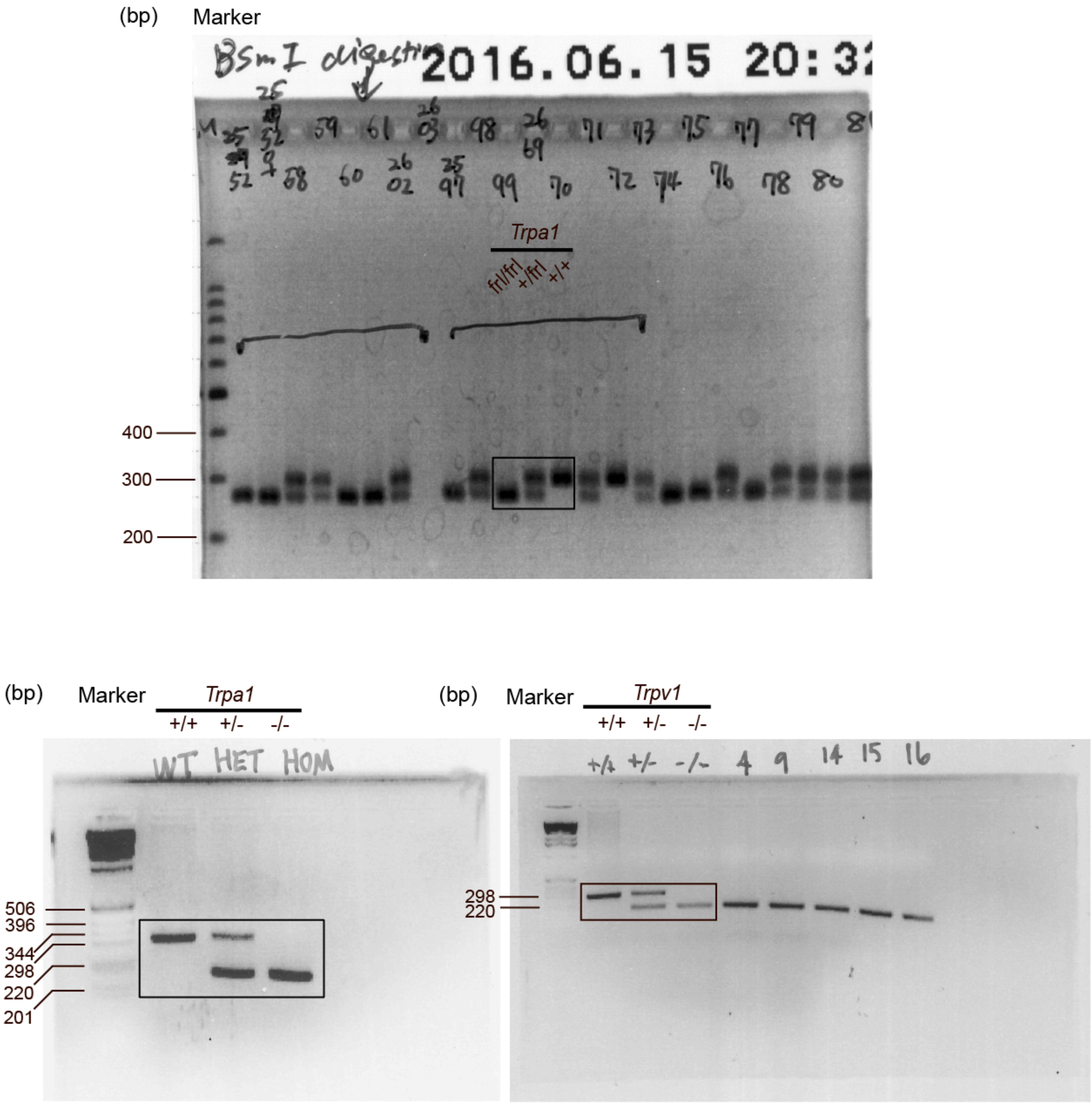

Supplementary Figure 12

Supplementary Figure 12. Full unedited DNA gel images for Supplementary Fig. 2a, 2b, 2c. Solid lines indicate areas shown in the Supplementary Figure.

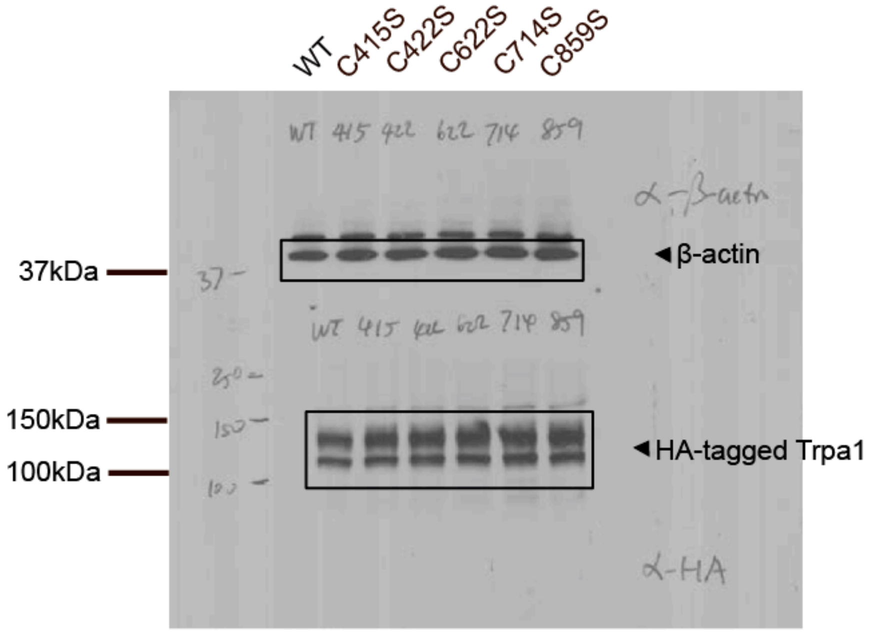

Supplementary Figure 13

Supplementary Figure 13. Full unedited DNA gel images for Supplementary Fig. 7f. Solid lines indicate areas shown in the Supplementary Figure.
