# Supplementary material for: Large-scale forward genetics screening identifies Trpa1 as a chemosensor for predator odor-evoked innate fear behaviors
Source: Nat Commun. 2018 May 23;9:2041. doi: 10.1038/s41467-018-04324-3 (PMC5966455; doi:10.1038/s41467-018-04324-3)
Supplement: Supplementary file 3 — Description of Additional Supplementary Files [file 41467_2018_4324_MOESM3_ESM.pdf]

Description of Additional Supplementary Files

File Name: Supplementary Movie 1
Description: A video comparing 2MT (1.05×10^{-4} mole)-evoked innate freezing behavior of wild-type and homozygous Trpa1$^{frl/frl}$ ENU mutant mice.

File Name: Supplementary Movie 2
Description: A video comparing TMT (2.98×10^{-4} mole)-evoked innate freezing behavior of Trpa1$^{+/-}$ and Trpa1$^{-/-}$ knockout mice.

File Name: Supplementary Movie 3
Description: A video comparing low concentration (1.05×10^{-6} mole) 2MT-evoked innate fear/defensive behaviors of Trpa1$^{+/-}$ and Trpa1$^{-/-}$ knockout mice.

File Name: Supplementary Movie 4
Description: A video comparing snake skin-evoked innate fear-like/defensive behaviors of Trpa1$^{+/-}$ and Trpa1$^{-/-}$ knockout mice.
